# The RUS1 (ROOT UVB SENSITIVE 1) Protein Is Required for Cold Resistance in *Chlamydomonas reinhardtii*

**DOI:** 10.3390/cells15080670

**Published:** 2026-04-10

**Authors:** Yulong Wang, Du Cao, Kangning Guo, Tingting You, Penghao Yang, Xiaobo Li

**Affiliations:** 1 College of Life Sciences, Zhejiang University, Hangzhou 310058, China; 2 State Key Laboratory of Gene Expression, School of Life Sciences, Westlake University, Hangzhou 310030, China; 3 Zhejiang Key Laboratory of Low-Carbon Intelligent Synthetic Biology, Westlake University, Hangzhou 310030, China; 4 Institute of Biology, Westlake Institute for Advanced Study, Hangzhou 310030, China

**Keywords:** *Chlamydomonas reinhardtii*, cold acclimation, pooled screening, ROOT UVB SENSITIVE

## Abstract

**Highlights:**

**What are the main findings?**

- Through pooled screening, 783 genes implicated in cold acclimation in *C. reinhardtii* were identified.
- CrRUS1 promotes cold acclimation in *C. reinhardtii* in a light-dependent manner.

**What are the implications of the main findings?**

- These findings expand our knowledge of cold acclimation genes in photosynthetic organisms and provide a foundation for cold stress research in land plants.
- The discovery of RUS provides a foundation for exploring how light signals specifically regulate cold acclimation in photosynthetic organisms.

**Abstract:**

Low temperature critically influences cellular metabolism by impairing processes such as membrane fluidity, enzyme activity, and protein folding. However, the comprehensive genetic landscape and regulatory mechanisms governing cold acclimation remain poorly understood. Here, we performed high-throughput, pooled genetic screening in the model alga *Chlamydomonas reinhardtii* (*C. reinhardtii*) to identify genes essential for cold acclimation. Our screening revealed numerous candidate genes implicated not only in early cold response pathways but also in core cellular processes, including DNA dynamics, protein homeostasis, metabolic regulation, and substrate transport. Notably, we identified a member of the RUS (ROOT UVB SENSITIVE) family, encoding a conserved DUF647 domain protein, designated CrRUS1. CRISPR-generated *rus1* mutant alleles in *C. reinhardtii* display a phenotype consistent with our screening: the mutants did not exhibit any visible growth defects, but show severe growth defects at low temperature. Interestingly, the cold-induced phenotypic changes in *rus1* can be reversed by dark conditions, suggesting that CrRUS1 likely promotes cold acclimation in *C. reinhardtii* through a light-dependent pathway. Our work provides novel genetic resources and mechanistic insights into cold acclimation in *C. reinhardtii*, with potential translational relevance for enhancing cold tolerance in crop species.

## 1. Introduction

Temperature is a critical factor that governs the structure and function of biological molecules and regulates virtually all vital reaction processes in vivo. Although temperature fluctuates naturally through diurnal and seasonal cycles, the increasing frequency of extreme and unpredictable weather events severely disrupts organismal metabolism and can lead to mortality [1,2]. For example, low temperature decreases the fluidity of cell membranes and impairs the functions of membrane proteins, thereby affecting substance transport and intercellular signaling [3,4,5]. Within the cell, low temperature leads to incorrect protein folding and decreases protein biosynthesis efficiency, compromising protein functionality. Simultaneously, it also alters DNA topology and RNA structure, further disrupting normal gene expression [6,7,8,9].

Sessile plants experience temperature changes passively as the ambient environment changes, with low temperatures frequently and extensively affecting key biological processes such as cell division, development, photosynthesis, and crop yield [7,10,11,12]. To cope with such stress, plants have evolved diverse adaptation mechanisms, including remodeling membrane lipid composition, accumulating cryoprotective metabolites or osmolytes, enhancing antioxidant systems, modulating protein folding chaperones or degradation enzymes, and entering dormancy [9,13,14,15,16]. Notably, plants can acquire enhanced tolerance to more severe low-temperature stress following a period of cold acclimation [17], implying that cold or chilling (0–15 °C), a more common and often preliminary stress, plays a critical role in preparing plants for subsequent freezing conditions (below 0 °C) [10,18]. Therefore, investigating cold acclimation mechanisms holds promise for improving freezing tolerance and informing the genetic improvement of crops.

Nevertheless, our understanding of the genetic basis of cold acclimation remains incomplete. Current knowledge is largely limited to a set of cold responsive genes and a small number of regulatory factors, while the mechanisms through which organisms alter gene expression and physiological metabolism to adapt to cold stress are still not fully understood [7,14,19,20,21,22]. To address these gaps, high-throughput genetic screening represents a powerful strategy for identifying mutants and genes involved in cold stress response [22,23]. Given the substantial resources and labor required for analogous studies in terrestrial plants, the unicellular green alga *C. reinhardtii* serves as an excellent model organism for systematically dissecting cold-related genes and acclimation mechanisms in photosynthetic organisms [24,25].

*C. reinhardtii* is a eukaryotic photosynthetic organism that shares fundamental cellular structures and metabolic functions with terrestrial plants [26,27,28]. Importantly, as a model organism in plant research, it exhibits conserved cold stress response mechanisms and is amenable to established high-throughput genetic tools [29,30,31,32,33]. Given that previous high-throughput cold screening efforts in *C. reinhardtii* have not yielded optimal results [32]; in this study, we newly constructed a large-scale mutant library of *C. reinhardtii* and identified classes of genes involved in cold acclimation through systematic screening.

*C. reinhardtii* is a haploid photosynthetic organism in which random insertion of exogenous resistance genes can lead to insertional inactivation of endogenous genes, resulting in corresponding phenotypic changes in the transformants. LEAP-Seq is a high-throughput technique for identifying exogenous DNA insertion sites [24,34]. This method employs biotinylated primers to perform unidirectional extension from within the resistance gene, followed by enrichment of the extension products, adaptor ligation, exponential PCR amplification, and next-generation sequencing. Ultimately, this approach associates the barcodes located within the resistance gene cassette with their corresponding insertion loci.

These findings are expected to substantially advance our understanding of cold acclimation mechanisms in photosynthetic organisms.

## 2. Materials and Methods

### 2.1. Algal Strains and Cell Culture

The *C. reinhardtii* strain CC-5325 (also known as CMJ030 or CC-4533) was obtained from the Chlamydomonas Resource Center (CRC (St. Paul, MN, USA); http://www.chlamycollection.org (Accessed: 12 November 2024)) and used as the background strain. Cells were grown in Tris-Acetate-Phosphate (TAP) liquid medium under continuous light (~60 μmol photons m^−2^ s^−1^) at 22 °C or 10 °C, with shaking at 120 rpm. For solid medium culture, cells were arrayed into 384-colony array and maintained or screened under the same conditions used for liquid experiments.

### 2.2. Mutant Library Construction and Screening

Mutants were generated by electroporation using a NEPA21 electroporator (Nepa Gene Co., Ltd., Ichikawa-City, Japan) as previously described [33,35,36], with the following modifications. Following electroporation, cells were transferred into 1 L of recovery buffer (TAP medium containing 40 mM sucrose) and kept in the dark at 22 °C with shaking for 24 h. Paromomycin was then added to a final concentration of 5 mg/L, and cells were cultured for an additional day. Subsequently, cells were transferred to light and grown for 5 days. At the end, cells were diluted 1:5 into fresh TAP medium containing no paromomycin and incubated for one day at 22 °C with shaking. The resulting pooled mutant population, designated CK1, served as the primary mutant library.

The CK1 library was divided into two portions. One portion was inoculated to an OD_750_ of 0.05 in 100 mL TAP medium and cultured continuously for two days at 22 °C under ~60 μmol photons m^−2^ s^−1^ light intensity with shaking. During this period, one dilution step was performed; the resulting culture was harvested as sample CK2. The other portion was inoculated to OD_750_ of 0.1 in 100 mL TAP medium and cultured continuously for four days at 10 °C under the same light intensity with shaking. After screening, these cells were collected as sample S10 and stored at −80 °C. A subset of the S10 culture was also subjected to recovery under the same conditions (22 °C) used for the transition from CK1 to CK2, yielding an additional sample designated S10R.

### 2.3. DNA Extraction, Barcode PCR, and LEAP-Seq

Cells from each sample were pelleted, flash-frozen in liquid nitrogen and stored at −80 °C. Total genomic DNA was extracted as previously reported [32]. The final pellet was air-dried and resuspended in 50 μL nuclease-free water. Finally, DNA concentration was measured using a Qubit fluorometer (Invitrogen, Carlsbad, CA, USA) and adjusted to 100 ng/μL.

Barcode PCR and LEAP-Seq were performed following established protocols [25,32]. For the LEAP assay, samples CK1 and S10R were used to identify the gene corresponding to each barcode. Primer sets 3′P2/3′P3-i3 (for CK1) and 3′P2/3′P3-i4 (for S10R) were employed. Differently, a universal and shorter LEAP-Seq adapter was used (Table S3), and the final exponential amplification step was increased to (10 + 18) cycles. For barcode quantification, barcode PCR was performed using sample-specific primer sets: 3′R1-1/3′R2-index2 (CK1), 3′R1-1/3′R2-index3 (CK2), 3′R1-3/3′R2-index4 (S10), and 3′R1-3/3′R2-index5 (S10R).

### 2.4. Data Analysis

For RNA-seq, ~4 × 10^7^ cells were harvested per sample. Total RNA was extracted using RNeasy Plus Mini Kit, and sequencing libraries were constructed and sequenced on an xplus platform (PE150) at Novogene Co., Ltd. (Beijing, China). 

Following quality control of raw RNA-seq FASTQ files with fastp, reads were aligned to the *C. reinhardtii* v5.6 reference genome using HISAT2 (v2.0.5) [37]. The resulting SAM files were sorted with SAMtools (v1.23.1), and gene-level read counts were obtained using featureCounts. Differential expression analysis between two conditions was performed using DESeq2 (v1.50.2). Genes with an adjusted *p*-value < 0.05 and a log_2_-based fold change greater than 1 or less than −1 were considered significantly regulated.

For the LEAP-Seq experiment, the methods and procedures followed the previous protocol [25]. All flanking sequences of LEAP-Seq were aligned to the *C. reinhardtii* v5.6 reference genome using BLAST (https://phytozome-next.jgi.doe.gov/blast-search (Accessed: 16 August 2025)). Insertion sites were annotated according to the following criteria: hits were categorized as ‘5′ UTR’, ‘exon’, ‘intron’, ‘3′ UTR’, ‘potential promoter’ (within 1 kb upstream of a transcription start site), or ‘intergenic.’ 

### 2.5. Lipid Extraction and Analysis

Frozen samples were immediately homogenized with 800 μL of lipid extraction solvent (methanol:chloroform:formic acid = 20:10:1, *v*/*v*). After adding 400 μL of lipid extraction buffer (0.4 M H_3_PO_4_, 1 M KCl), the mixture was vortexed for 30 s and centrifuged (3000× *g*, 5 min). The lower organic phase was transferred to a new tube, dried under N_2_. For separation, the lipid concentrate was subjected to thin-layer chromatography (TLC) using a chloroform:methanol:acetic acid:water (75:13:9:3, *v*/*v)* solvent buffer. Individual bands of each sample were scraped from the air-dried, stained plate.

For fatty acid methyl ester (FAME) derivation, each sample was spiked with 50 μL of C17:0 in methanol (internal standard) and 700 μL of BF_3_-methanol, then heated at 75 °C for 30 min. After cooling, 1 mL each of water and n-hexane were added successively. The mixture was vortexed vigorously and centrifuged (2000× *g*, 5 min). The colored lower phase was transferred in a fume hood to a hexane-rinsed tube and dried under N_2_. Finally, the residue was dissolved in 100 μL n-hexane, and transferred to a GC vial for analysis.

### 2.6. Mutants Generation by CRISPR-Cas9

In *C. reinhardtii*, gene editing techniques have been widely adopted, and we followed established protocols with minor modifications [38,39]. Briefly, Cas9-single guide RNA (sgRNA) ribonucleoprotein (RNP) assembled in vitro were introduced, along with donor DNA containing antibiotic resistance cassette, into *C. reinhardtii* cells by electroporation. Recombinant Cas9 protein was expressed and purified in vitro as previously described [40]; sgRNAs were designed using CRISPR-P 2.0 (http://crispr.hzau.edu.cn/cgi-bin/CRISPR2/CRISPR (Accessed: 17 October 2025)) [41] and chemically synthesized by GenScript. For subsequent transformation and screening, donor DNA fragment containing the paromomycin-resistance gene was amplified from the plasmid pMJ016c [24] via PCR and diluted to 200 ng/μL. Sequences of all sgRNAs and primers used for donor DNA amplification are listed in Table S3.

For transformation, RNP complexes were assembled in vitro by incubating sgRNAs with Cas9 protein in NEBuffer 3.1 (B7203, NEB) at 37 °C for 15 min, in a final volume of 10 µL. Meanwhile, CC5325 cells harvested at the logarithmic growth phase were first subjected to a 30-min heat treatment at 40 °C, followed by collection via low-speed centrifugation. The cells were washed once with recovery buffer (TAP with 2% *w*/*v* sucrose) and resuspended to a density of 2 × 10^8^ cells/mL. Both the RNP complexes (10 µL) and donor DNA (5 µL) were co-electroporated into the cells (110 µL) using 0.2 cm-gap cuvette (EC-002S, Nepa Gene Co., Ltd., Ichikawa-City, Japan) and BTX Gemini X2 electroporation system (Harvard Bioscience company, Holliston, MA, USA) at 350 V, 25 Ω, and 600 μF. Immediately, the cuvettes were incubated at 16 °C for 1 h, and transferred to recovery buffer for one day [42]. After one week’s selection on TAP plates containing 25 mg/L paromomycin, transformants became clearly visible and were subsequently used for genotypic and phenotypic validation.

### 2.7. Measurement of Chlorophyll, F_v_/F_m_ and Growth

For chlorophyll content measurement, *C. reinhardtii* cells at a specified density were resuspended in 80% acetone. After incubation in the dark for 10 min, the suspension was centrifuged to remove debris. The absorbance of the supernatant was then measured at 647 nm and 664 nm, and the chlorophyll content per cell was calculated according to the published method [43].

Chlorophyll fluorescence parameters were measured as previously described [44]. Briefly, cells were dark-adapted for 20 min. Subsequently, chlorophyll fluorescence readings were recorded and output for each sample using an Imaging-PAM system (Walz, Heinz Walz GmbH, Bavaria, Germany). The maximal quantum efficiency of photosystem II (*F_v_*/*F_m_*) was calculated as (*F_m_* − *F_o_*)/*F_m_*, where *F_o_* and *F_m_* represent the minimum and maximum chlorophyll fluorescence yields, respectively [45]. Data were finally processed with GraphPad Prism software (v10.1.2).

For growth assay, log-phase cells were concentrated to 2 × 10^7^ cells/mL. A 10 µL aliquot of the suspension was spotted onto TAP solid plates, followed by serial dilutions. Subsequently, plates were incubated either at 22 °C for 3 days (control) or at 14 °C for 16 days (cold stress) under ~60 µmol photons m^−2^ s^−1^ light or darkness, before photographic documentation.

### 2.8. Mutants Identification and Validation

After antibiotic selection, transformants were picked robotically and arrayed onto TAP plates containing 25 mg/L paromomycin in a 384-colony format. After four days of growth, colonies were robotically transferred to two copies on TAP plates and incubated at 22 °C or 10 °C respectively for another four days.

Mutants with predominant phenotype changes at 10 °C but general growth at 22 °C were selected for further validation by PCR. At the same time, at least one mutant without phenotype changes was selected as control.

Diagnostic PCR for each mutant gene was performed using primers designed to flank the gRNA target site by about 1 kb on each side. Compared with the control, the PCR product of truth mutants was theoretically predicted to be approximately 2 kb larger, which is consistent with the donor DNA insertion. The sequences for the primers used in genotyping PCR are provided in Table S3.

### 2.9. Phylogenetic Analyses

Homologous sequences were identified by performing a DIAMOND (v2.0.9.147) blastp search against the UniProt protein database (release 29 October 2023) using the parameters --evalue 1e-5 --id 40 [46]. The resulting hits for Cre03.g148150 were then subjected to clustering with CD-HIT (v4.8.1) at a 70% identity threshold (-c 0.70). The clustered sequences were aligned using MAFFT (v7.505), followed by phylogenetic tree construction with IQ-TREE2 (v2.2.2.6). ModelFinder, built into IQ-TREE2, was used for automated model selection, which identified JTT + I + R10 as the optimal substitution model according to the Bayesian Information Criterion (BIC) [47]. Branch support was evaluated with 1000 replicates of the Ultrafast Bootstrap.

## 3. Results

### 3.1. Mutant Library Construction and Screening

**Figure 1:**
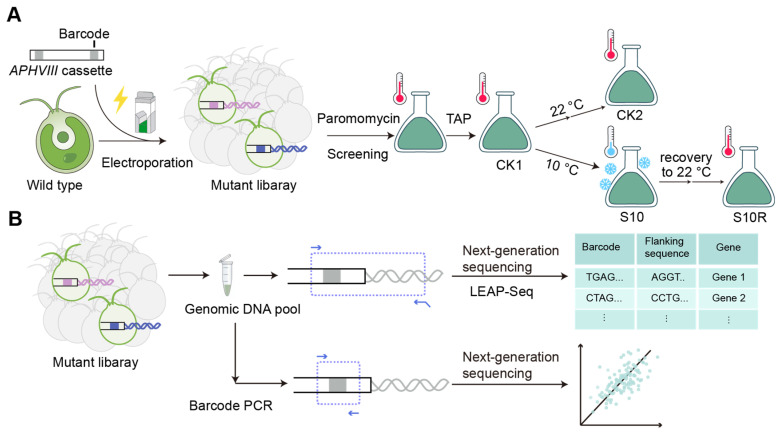
Workflow of cold tolerance screening: (**A**) Mutant library construction and cold treatment. DNA cassettes were PCR amplified using pMJ016c as the template and diluted to 5 ng/μL. The mutant pool (CK1) was subjected to two successive dilution steps to obtain sample CK2. For cold screening, the mutant pool was incubated at 10 °C for 4 days to obtain sample S10. After two consecutive rounds of recovery cultures from S10, sample S10R was collected. (**B**) Analysis of barcode abundance and identification of corresponding genes by LEAP-Seq. White boxes indicate cassette backbone; colored boxes indicate unique barcode sequence; spiral lines indicate flanking genomic DNA.

To achieve high-throughput screening of the *C. reinhardtii* genome, we randomly integrated DNA cassettes into the genome via electroporation to disrupt the genes at their insertion sites [25]. After transformation and recovery, the cells were transferred to medium containing paromomycin and grown under continuous illumination. Upon visible growth, indicated by a green coloration, the resulting mutant library was diluted with TAP medium without antibiotic (CK1) and divided into two portions. One portion was transferred into new TAP medium at 22 °C (RT, room temperature) to serve as a control (CK2). The other portion was cultured in new TAP medium but subjected to low-temperature (10 °C) treatment (S10). Subsequently, a subset of these cold-stressed cells was returned to 22 °C (S10R) to assess the recovery capacity of individual mutants (Figure 1A).

During the experimental process, samples from the initial mutant library pool, the control group, and the experimental group were separately collected (the screening process is carried out once). Following total genomic DNA extraction, a portion of the genomic DNA from each sample was used for barcode PCR and subsequent Next-generation sequencing to determine the relative abundance of each mutant or barcode within the respective sample. By comparing across different samples, we could precisely track changes in the abundance of each barcode before and after cold stress treatment. Meanwhile, we selected the initial mutant library pool and the cold stress recovery sample to perform LEAP-Seq followed by Next-generation sequencing [24]; analysis of the LEAP-Seq data allowed us to establish the correspondence between each barcode and the genomic insertion site of its respective cassette, thereby identifying the mutated gene (Figure 1B).

### 3.2. 783 Genes Implicated in Cold Acclimation in C. reinhardtii

**Figure 2:**
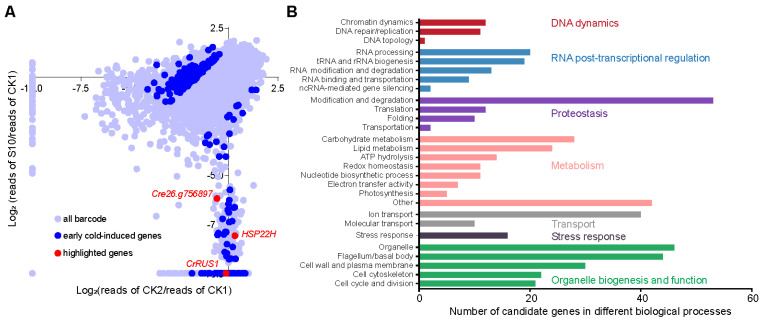
High-throughput screening for cold stress acclimation in *C. reinhardtii*: (**A**) Scatter plot depicting the relative abundance of individual barcodes under control versus cold-stress conditions. The x- and y-axes show the log_2_-based fold change in CK2/CK1 and S10/CK1 ratios, respectively. An offset of 0.001 was added to all ratio values prior to log_2_ transformation. Each dot in the figure represents a barcode, with dark blue dots denoting early cold-induced genes identified through LEAP-Seq, while red dots indicate genes highlighted in this study. The data are provided in Table S1. (**B**) Functional profiling of candidate genes identified in the screen. The analysis includes only genes that have functional annotation.

Based on data analysis of barcode PCR, barcodes with a read count ≥50 in the CK1 sample were selected for subsequent analysis. After filtering, a total of 37,389 barcodes were obtained. We assessed the changes in barcode abundance before and after cold stress by calculating the CK2/CK1 and S10/CK1 ratios (Figure 2A). Through threshold-based screening (the ratio (S10/CK1)/(CK2/CK1) greater than 3 or less than 0.3), 1794 barcodes showed substantial changes in abundance. Additionally, according to data analysis of LEAP-Seq, 866 barcodes were successfully mapped to their corresponding genomic insertion sites, representing 783 candidate genes (Table S1).

As previously reported, cold stress-responsive genes are induced within minutes. Studies have identified 3471 differentially expressed genes as early as one hour of cold exposure; these genes are involved in various biological processes, including protein synthesis, cell cycle, and protein kinase-based phosphorylation [48]. Consistent with this, comparative analysis revealed that 221 of the candidate genes belong to early cold-induced genes. Notably, this subset includes genes encoding the Calcium-dependent lipid-binding (CaLB domain) family protein (Cre01.g015500), the C2C2_GATA transcription factor (Cre03.g146267) and the C2C2_CO-like transcription factor (Cre12.g521150) (Table S1). Interestingly, these 221 candidate genes could be classified into two main groups based on our screening. Mutants in the first group exhibited a constitutive growth defect under normal conditions, which were alleviated under cold stress. In contrast, mutants in the second group grew normally under control conditions but displayed severe growth inhibition specifically under cold stress (Figure 2A). These findings align with our expectations that most cold-induced genes participate in fundamental biological processes, and mutations in these genes generally reduce cellular growth capacity. Notably, numerous genes appear to function specifically within cold stress response pathways and play essential biological roles in this process. While both groups are relevant, we focus primarily on genes in the second group, as they represent critical targets for future research aimed at elucidating gene regulatory networks involved in cold acclimation in photosynthetic organisms.

It is well established that low temperature stress extensively impacts cellular structure and function. Conversely, cellular components such as membranes, proteins, and nucleic acids can themselves act as thermosensors, perceiving ambient temperature changes and initiating the cold stress response [6,49,50]. As expected, functional analysis revealed that the candidate genes span a broad spectrum of biological processes, including proteostasis, cellular metabolism, substance transport, and organelle biogenesis (Figure 2B). For instance, heat shock proteins (HSPs) are a widely studied class of molecular chaperones that facilitate protein folding and have been reported to accumulate under low-temperature stress [51,52]. In our screening, we identified a member of the *HSP22* subfamily of the HSPs family, Heat Shock Protein 22H (HSP22H, Cre07.g318600). Consistently, the barcode corresponding to *hsp22h* mutant became nearly undetectable under cold stress (Figure 2A).

### 3.3. CrRUS1 Promotes Cold Acclimation of C. reinhardtii

**Figure 3:**
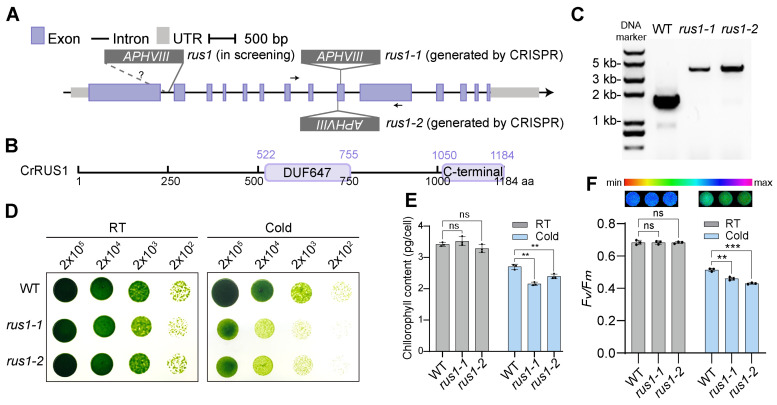
Molecular and phenotypic characteristics of the *rus1*: (**A**) Gene model of *CrRUS1*. A schematic diagram of the insertion site of *rus1* mutant lines, with arrows indicating primer locations. Primer sizes are not drawn to scale. (**B**) Functional domain annotation of CrRUS1. (**C**) DNA gel showing the PCR products of genotyping identification. (**D**) Phenotypic verification of the *rus1* mutant. WT, wild type. Cells were grown at 22 °C for 3 days (RT) or at 14 °C for 16 days (cold). (**E**) Chlorophyll content and (**F**) *F_v_*/*F_m_* measurement of cells under different temperatures. The cells were treated with cold (10 °C) for 2 days. In this representation, the parameter’s maximum and minimum values are denoted in purple and red, respectively. Statistical significance versus the wild-type (WT) was determined using Welch’s *t* test (ns, not significant, ** *p* < 0.01, *** *p* < 0.001); values represent the mean of three biological replicates.

Among the candidate genes identified in the screen, we focused on an evolutionarily conserved gene Cre03.g148150 (designated *CrRUS1*), which encodes a protein containing an uncharacterized conserved domain DUF647 and a specific C-terminal domain (Figure 3A,B). The barcode corresponding to the *rus1* mutant exhibited comparable abundance under normal conditions but became nearly undetectable under both the cold-stress (S10) and recovery (S10R) samples (Figure 2A and Table S1).

To validate screening results, we specifically generated *rus1* knockout mutants using CRISPR-Cas9. During the repair process following Cas9-induced DNA double-strand breaks, donor DNA may be inserted at the target site, resulting in insertional inactivation of the target gene. The PCR products of the mutants were 2 kb larger than those of the wild-type control, corresponding to the length of the donor DNA, confirming that both selected mutants were confirmed as correct insertional knockout mutants and chosen for phenotypic analysis (Figure 3A,C, Supplementary Figure S1). As expected, following cold treatment, the *rus1* mutants showed a severe growth defect, while their growth rate under normal condition was comparable to that of the wild-type (Figure 3D). To eliminate the possible effects of different low temperatures on the phenotype of the *rus1* mutant, we compared the growth differences between *rus1* and the wild type (WT) at 14 °C and 10 °C (Supplementary Figure S2). We found that *rus1* exhibited the same growth defects under both low-temperature conditions. These results are consistent with the high-throughput screening data for *rus1*, further confirming the reliability of our screening approach.

To rule out potential off-target effects, we designed a new gRNA and performed additional knockout experiments. Notably, *rus1* mutants generated using different gRNAs exhibited similar phenotypic changes, and compared with the wild-type (WT) control, the *rus1* mutant displayed obvious growth defects under cold conditions (Supplementary Figure S3A,B).

In parallel, we conducted a more detailed phenotypic analysis of the *rus1* mutants. Similarly to the growth phenotype, no obvious photosynthesis-related defects were observed under normal conditions (Figure 3E,F). Under cold stress, however, the mutants exhibited significantly reduced chlorophyll content and *F_v_*/*F_m_*, with decreases of approximately 14% and 18%, respectively.

### 3.4. Transcriptomic Characterization of rus1

**Figure 4:**
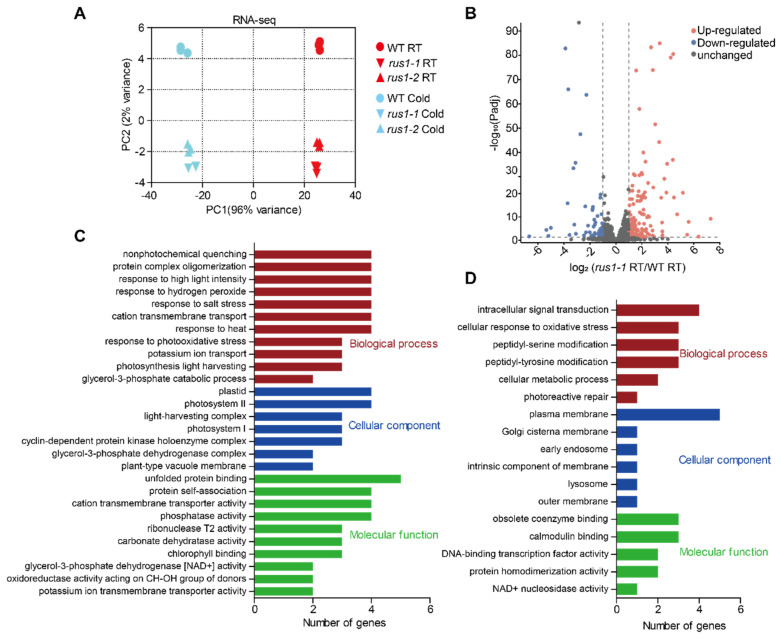
Differentially expressed genes and Gene Ontology analysis of *rus1*: (**A**) Principal component analysis (PCA) based on transcriptome data. (**B**) *CrRUS1*-dependent transcriptional changes under normal temperature. (**C**,**D**) GO term enrichment analysis of genes up-regulated (**C**) and down-regulated (**D**) in *rus1* under normal temperature. The experiment was performed with three independent biological replicates.

To elucidate the molecular mechanism by which CrRUS1 promotes cold adaptation in *C. reinhardtii*, we analyzed changes in the gene expression profile of the mutant under early cold stress and normal conditions. Principal component analysis (PCA) revealed that temperature regime was the primary driver of transcriptional differences between wild-type and mutant strains, while genotype-driven variations played a secondary role (Figure 4A). Additionally, we hypothesized that factors contributing to the cold-specific phenotype of the *rus1* mutant may originate prior to cold exposure. Based on these observations, we prioritized the analysis of gene expression changes in *rus1* under normal growth conditions. Transcriptomic analysis revealed 209 upregulated genes and 120 downregulated genes in the *rus1* mutant relative to the wild type under normal conditions (Figure 4B). Notably, more than half of these differentially expressed genes (DEGs) are cold-responsive, suggesting their dysregulation may underpin the cold-sensitive phenotype of *rus1*. Functionally, upregulated DEGs were predominantly enriched in abiotic stress responses and photosynthesis, in contrast to downregulated DEGs, which were mainly involved in signal transduction, protein modification, and protective systems (Figure 4C,D). Differently, cold stress triggered extensive transcriptomic reprogramming in the *rus1* mutant (Table S2), and the DEGs were primarily enriched for functions related to cytoskeleton formation and organization (Supplementary Figure S4). Furthermore, a large subset of these genes were of unknown function, representing potential novel players in cold acclimation in *C. reinhardtii*.

Although multiple biomolecules are known to function as thermosensors in cold signaling, the most immediate and widespread impact of low temperature is a reduction in membrane fluidity, which perturbs membrane-associated processes. Given this, and to elucidate the molecular function of CrRUS1 in cold acclimation, we specifically analyzed the polar lipid and fatty acid profiles of the *rus1* mutant. Strikingly, no statistically significant difference was observed in the relative abundance of major membrane lipid classes between the wild-type and the *rus1* mutant (Supplementary Figure S5). Regarding total fatty acids in the extract, no significant differences were observed between the mutant and wild-type strains in any fatty acid species, although low temperature led to a marked decrease in 18:1^Δ9^ content and a slight reduction in 18:2^Δ9,12^ levels in *C. reinhardtii* [30]. The results suggest that CrRUS1 promotes cold acclimation in *C. reinhardtii* through an unknown mechanism, rather than via membrane remodeling.

### 3.5. Function Prediction of CrRUS1

**Figure 5:**
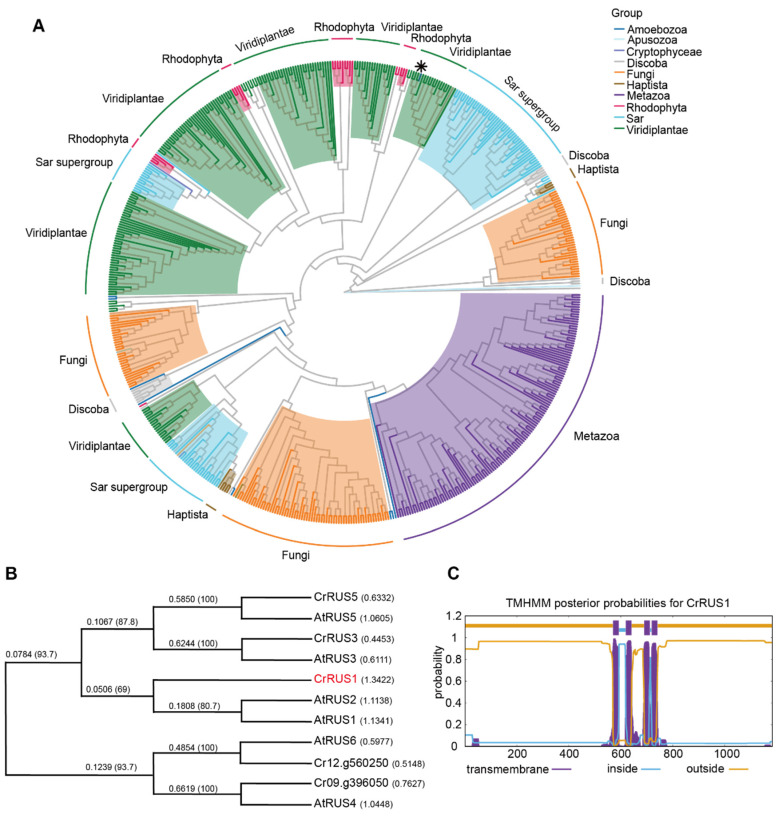
Protein sequence alignment and structure prediction of CrRUS1: (**A**) Maximum-likelihood phylogeny of the RUS family. The star indicates CrRUS1 in *C. reinhardtii*. (**B**) RUS protein sequence alignment in *Chlamydomonas* and *Arabidopsis*. The protein sequences were aligned using the CLUSTALW online tool (https://www.genome.jp/tools-bin/clustalw (Accessed: 13 December 2025)) with subsequent data export. The Multiple Alignment Parameters were set to their default values, and the Identity matrix (ID) was selected as the Weight Matrix for protein sequences. (**C**) Prediction of the transmembrane structure of the RUS protein. Data were obtained from online analysis using TMHMM-2.0 (https://services.healthtech.dtu.dk/services/TMHMM-2.0/ (Accessed: 26 February 2026)).

The RUS protein family comprises multiple members across most eukaryotic species, including animals, fungi, land plants and algae [53,54]. Phylogenetic analysis revealed that within Viridiplantae, DUF647 domain of RUS proteins clusters into six distinct clades (Figure 5A). Among them, five clades are closely related, and the majority of all clades are associated with corresponding Rhodophyta lineages. Similarly, in fungi, DUF647 forms at least three separate clades, and one of the clades is closely related to the metazoan lineage. These results suggest that the ancestors of photosynthetic eukaryotes and fungi underwent multiple rounds of gene expansion and functional divergence of DUF647-containing proteins during evolution. Conversely, DUF647 exhibits high conservation within the metazoan lineage, forming only a single major clade. It is noteworthy that current evidence indicates that the ortholog of RUS proteins in animals corresponds to RUS3 in plants, though its function is still unknown [53].

In *A. thaliana*, there are six *RUS* genes (*RUS1*-*6*) in the genome: *RUS1* and *RUS2* play critical roles in early seedling development and an embryo-lethal phenotype in *rus6* mutants; the functional roles of *RUS3*, *RUS4*, and *RUS5* in *A. thaliana* remain largely uncharacterized [53,54,55,56]. In *C. reinhardtii*, BLAST analysis identified five proteins containing the DUF647 domain: Cre03.g148150 (RUS FAMILY MEMBER 1), Cre03.g187650 (CrRUS5), Cre09.g396050 (RUS FAMILY MEMBER 1), Cre12.g560250 (Uncharacterized conserved protein), and Cre16.g671150 (CrRUS3). Phylogenetic analysis based on protein sequence alignment revealed that CrRUS1 clusters with AtRUS1 and AtRUS2 from *A. thaliana*, while Cre12.g560250 and Cre09.g396050 show closer homology to AtRUS6 and AtRUS4, respectively (Figure 5B).

Structural analysis of CrRUS1 indicated that conserved DUF647 domain contains four transmembrane helices, and its spatial structure resembles that of ion transporters or solute carrier transporters (Figure 5C). Additionally, CrRUS1 contains a distinct structural domain at its carboxyl terminus (residues 1050–1184) (Figure 3B), which is primarily composed of α-helices and β-sheets and is connected to the DUF647 domain via a disordered region. This suggests that CrRUS1 may function as a transmembrane protein involved in intracellular substance transport [55,57] and DUF647 domain and/or C-terminal domain may participate in protein-protein interactions or regulate its structure and activity [53].

### 3.6. CrRUS1-Dependent Cold Acclimation in C. reinhardtii Is Light-Dependent

CrRUS1 shows higher sequence similarity to *A. thaliana* RUS1 and RUS2, and its predicted structure is more closely related to that of RUS2 (Figure 5B). Differently, previous studies showed that the *rus1* and *rus2* mutants exhibited marked developmental defects under white light, particularly UV-B, including stunted primary root growth and failure to form postembryonic leaves in *A. thaliana* [53,56]. In this study, however, the *rus1* mutant in *C. reinhardtii* did not display any obvious growth defects under normal conditions (light, 22 °C) (Figure 3D). These results imply that CrRUS1 does not participate in light-regulated growth processes under normal conditions.

**Figure 6:**
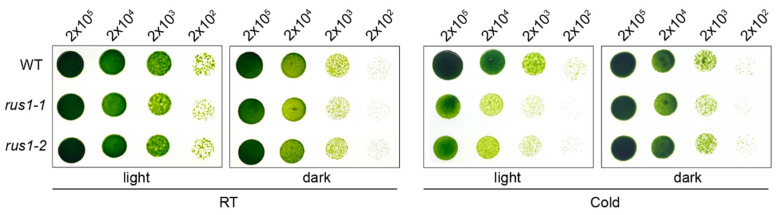
Growth phenotype of *rus1* under combined conditions. For experiments at RT, cells in the light group were grown for 3 days at 22 °C, whereas those in the dark group were grown for 7 days at 22 °C. Under cold condition, cells in the light group were grown for 16 days at 14 °C, whereas those in the dark group were grown for 30 days at 14 °C. The number of cells spotted per dilution is indicated above the lanes.

To further investigate the biological mechanism by which CrRUS1 promotes cold adaptation in *C. reinhardtii*, we examined the growth performance of the *rus1* mutant under cold/dark condition. Interestingly, the cold-induced defective phenotype of the *rus1* was reversed by darkness, as no obvious growth defect was observed under combined cold and dark conditions (Figure 6).

## 4. Discussion

*C. reinhardtii* has long served as a model organism for studying gene function and stress response mechanisms in photosynthetic eukaryotes [25,26,32]. However, the molecular processes underlying its acclimation to low temperature remain poorly understood. To address this gap, we constructed a genome-wide mutant library in *C. reinhardtii* and performed high-throughput screening to identify mutants defective in cold acclimation. Our analysis revealed 783 genes implicated in this process, which are associated with diverse biological processes, including protein processing and turnover, DNA dynamics, plasma membrane remodeling, and cellular metabolism (Figure 2B). Notably, over 200 of these candidate genes have previously been linked to early cold response in *C. reinhardtii*; and mutations in most of them may lead to decreased cellular growth capacity even under normal temperatures. Importantly, many candidate genes exhibit growth defects only under cold stress, providing a valuable genetic resource for future studies. Functional and regulatory characterization of these genes will not only advance our understanding of cold acclimation in photosynthetic organisms, but may also inform strategies for developing cold-tolerant crops.

In land plants, we identified 380 orthologs of the 783 candidate genes based on protein sequence alignment, and these are similarly involved in diverse biological processes in *A. thaliana* (Table S1). Future functional validation and studies of these 380 genes will not only expand our understanding of the gene regulatory networks underlying cold acclimation in land plants, but also help us compare the similarities and differences in cold adaptation between aquatic and terrestrial photosynthetic organisms. For instance, the HSP family genes mentioned above are a class of well-known genes involved in various stress responses and appeared in our cold screening results (e.g., *HSP22H*). The barcode corresponding to *hsp22h* exhibited similar abundance under normal temperature conditions, but its abundance was almost undetectable under cold conditions, suggesting that this gene may positively regulate cold acclimation in *C. reinhardtii*. The mechanism by which HSP22H or chaperone participates in cold acclimation in *C. reinhardtii* warrants further in-depth investigation. Another example, the protein ubiquitination pathway mediated by the RING/U-box family is known to play crucial roles in proteostasis in both *C. reinhardtii* and land plants [58,59], and has been implicated in cellular cold acclimation, likely through modulating protein homeostasis [48,60,61]. Consistent with this, our high-throughput screening and subsequent data analysis revealed that a mutant of the RING/U-box family gene Cre26.g756897 exhibited complete growth arrest under cold stress (Figure 2A). This gene is annotated as encoding an “Antifreeze protein” and its *A. thaliana* ortholog *DA2* is a well-characterized E3 ubiquitin ligase that negatively regulates seed and organ size [62,63]. Whether *DA2* also participates in cold acclimation in *A. thaliana* remains to be explored.

The RUS family is highly conserved across eukaryotes, with all members sharing a conserved DUF647 domain. *A. thaliana possesses six RUS genes;* among these, the functions of *RUS1*, *RUS2*, and *RUS6* have been partially characterized, while those of *RUS3*, *RUS4*, and *RUS5* remain largely unknown [53,54,57]. Protein sequence alignment confirmed that CrRUS1 is highly homologous to RUS1 and RUS2 (Figure 5B). However, in contrast to its homologs, *rus1* mutant shows undetectable growth defect under normal conditions and is insensitive to light in *C. reinhardtii*. More intriguingly, the growth defects of the *rus1* mutant under cold stress can be reversed under dark conditions, as *rus1* exhibited no obvious impairment under cold/dark conditions on TAP medium (Figure 3D and Figure 6). These results demonstrate that CrRUS1 possesses functions and signaling pathways that are completely different from those of AtRUS1/2 in *A. thaliana*. In *C. reinhardtii*, we also found that low temperature alone is insufficient to induce the growth defects in *rus1*. Instead, light exposure during cold stress maybe the critical trigger. The observed phenotype changes under cold likely stem from a combination of photosynthetic dysfunction—reflected in dysregulated expression of stress response genes and photosynthetic genes under normal conditions, along with reduced chlorophyll content and lower *F_v_*/*F_m_* ratios under cold stress (Figure 3 and Figure 4).

Previous studies in *A. thaliana* have shown that AtRUS2 localizes to the chloroplast and facilitates auxin transport through its interaction with the DUF647 domain of AtRUS1 [53,57]. Investigating the subcellular localization of CrRUS1 will not only help us gain a deeper understanding of its function but also enhance our understanding of the mechanism by which light signals regulate cold acclimation in photosynthetic organisms. In *C. reinhardtii*, although CrRUS1 was predicted to localize to mitochondria [64,65], it was not detected in mitochondrial proteomics datasets [66]. Due to the technical challenges of gene cloning in *C. reinhardtii* [67,68], attempts to clone the full-length Cr*RUS1* gene—which features a long genomic sequence (7635 bp, *C. reinhardtii* v5.6) and high GC content (68%)—failed. Currently, functional insights into CrRUS1 based on protein sequence and structural prediction indicate that it is likely a transmembrane protein, with its DUF647 domain forming a core transmembrane region (Figure 5C). In contrast to the conserved DUF647 domain, the C-terminal domain of CrRUS1 shows low conservation and appears protein-specific, suggesting a potential role in functional modulation through specific protein-protein interactions. Future studies on the CrRUS1-interacting proteins will help elucidate the molecular regulatory network involving CrRUS1, while also deepening our understanding of how light signals participate in regulating cold acclimation in *C. reinhardtii*.

The phenotypes of *rus1* under cold and dark conditions differed from those under cold and light conditions, suggesting that light signaling is involved in the cold acclimation of *C. reinhardtii*. Studies in land plants have suggested that both photoreceptors and the photosynthetic electron transport chain may participate in the perception or transmission of cold signals [69,70,71]. However, we have not yet determined which light-dependent pathways CrRUS1 uses to affect cold acclimation in *C. reinhardtii*, such as those involved in chloroplast functional integrity maintained by material transport, photosynthetic electron transport and its byproducts (ROS, photoinhibition, ATP/NADPH balance), photosynthetic products, or light signals mediated by different photoreceptors. Unfortunately, transcriptomic analysis did not detect differential expression of genes related to photoreceptors or photosynthesis. This may be attributed to the early time point of sampling (1 h of cold treatment), as our objective was to identify early response genes involved in the cold stress response mediated by CrRUS1. To elucidate the mechanism by which CrRUS1 and light signaling mediate cold acclimation, photoreceptor gene mutants, photosynthesis-related gene mutants, and/or their corresponding double mutants with *rus1* should be generated and characterized in future work.

## 5. Conclusions

In summary, this study employed high-throughput screening to systematically investigate genes involved in cold acclimation in *C. reinhardtii*. Through data analysis and protein sequence alignment, a total of 783 candidate genes were identified, corresponding to 380 orthologs in *A. thaliana*. Phenotypic and molecular characterization of the *rus1* mutant validated the reliability of the screening approach and revealed a role for CrRUS1 in promoting cold acclimation in *C. reinhardtii*. This work lays a foundation for further functional characterization of candidate genes and can contribute to a deeper understanding of the cold stress response mechanisms in photosynthetic organisms beyond green algae.

## Data Availability

The data supporting this study are available within the article and its Supplementary Materials. Further inquiries can be directed to the corresponding author.

## Supplementary Materials

The following supporting information can be downloaded at: https://www.mdpi.com/article/10.3390/cells15080670/s1, Figure S1: Sequencing results of the *rus1* mutant; Figure S2: Comparison of *rus1* phenotypes under different low temperatures; Figure S3: Characterization of the genotype and phenotype of the *CrRUS1*-gRNA1 and gRNA2 mutants; Figure S4: GO term enrichment analysis of DEGs in *rus1-1* under cold conditions; Figure S5: Relative abundance of relative fatty acid (FA) composition and major polar lipid classes. Table S1: Candidate genes identified under cold screening; Table S2: Gene expression profiles of WT and rus1 under 22 °C or 10 °C; Table S3: The list and sequence information of primers used in this study.
