# Supplementary material for: The RUS1 (ROOT UVB SENSITIVE 1) Protein Is Required for Cold Resistance in Chlamydomonas reinhardtii
Source: Cells. 2026 Apr 10;15(8):670. doi: 10.3390/cells15080670 (PMC13115044; doi:10.3390/cells15080670)
Supplement: Supplementary file 1 [file cells-15-00670-s001.zip › cells-4205001-supplementary.pdf]

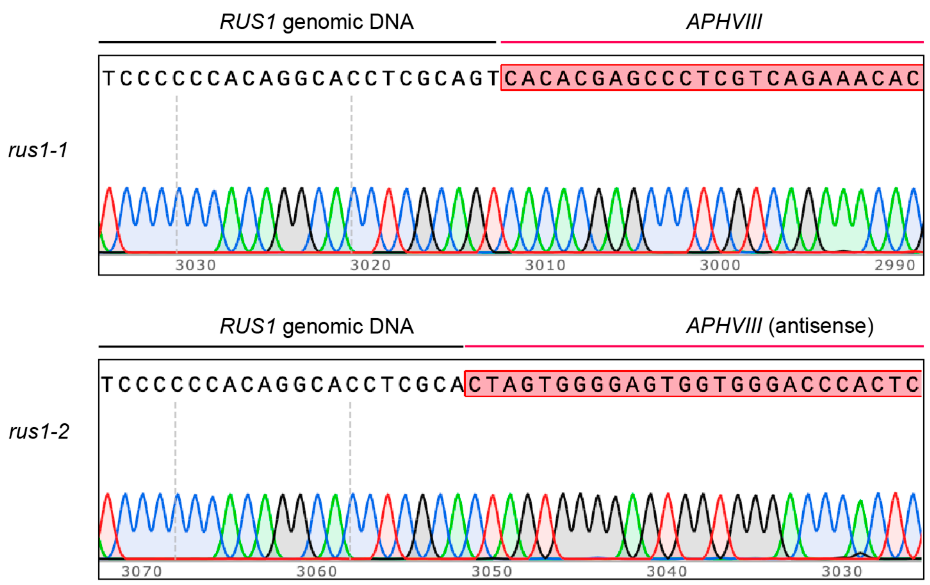

Supplementary Figure S1. Sequencing results of the *rus1* mutant. Sanger sequencing results of the Cr*RUS1* gene in the *rus1-1* and *rus1-2* mutants (data refer to Figure 3A).

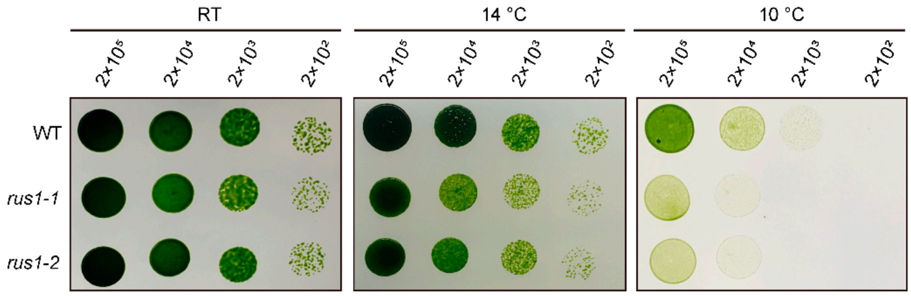

Supplementary Figure S2. Comparison of *rus1* phenotypes under different low temperatures. Cells were grown at RT (22 °C) for 3 days, and at 14 °C and 10 °C for 16 days.

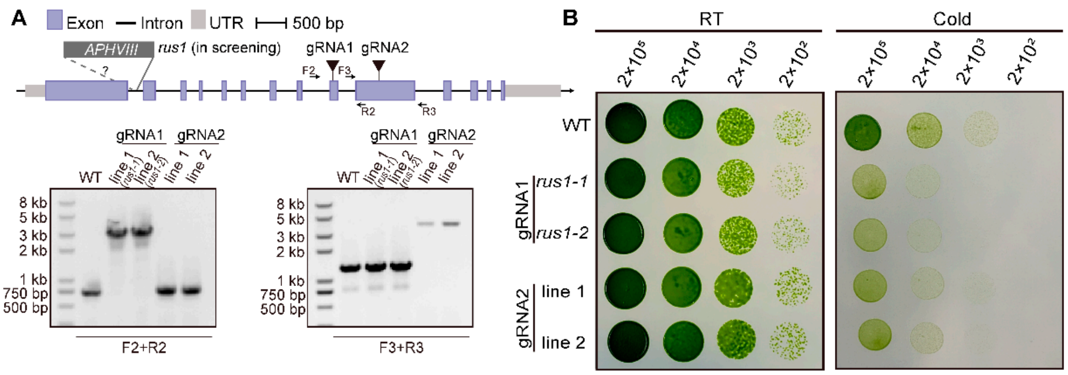

Supplementary Figure S3. Characterization of the genotype and phenotype of the *CrRUS1*-gRNA1 and gRNA2 mutants. (**A**) DNA gel showing the PCR products of genotyping identification. (**B**) Phenotypic verification of the *rus1* mutant. WT, wild type. Cells were grown at 22 °C for 3 days (RT) or at 10 °C for 16 days (cold).

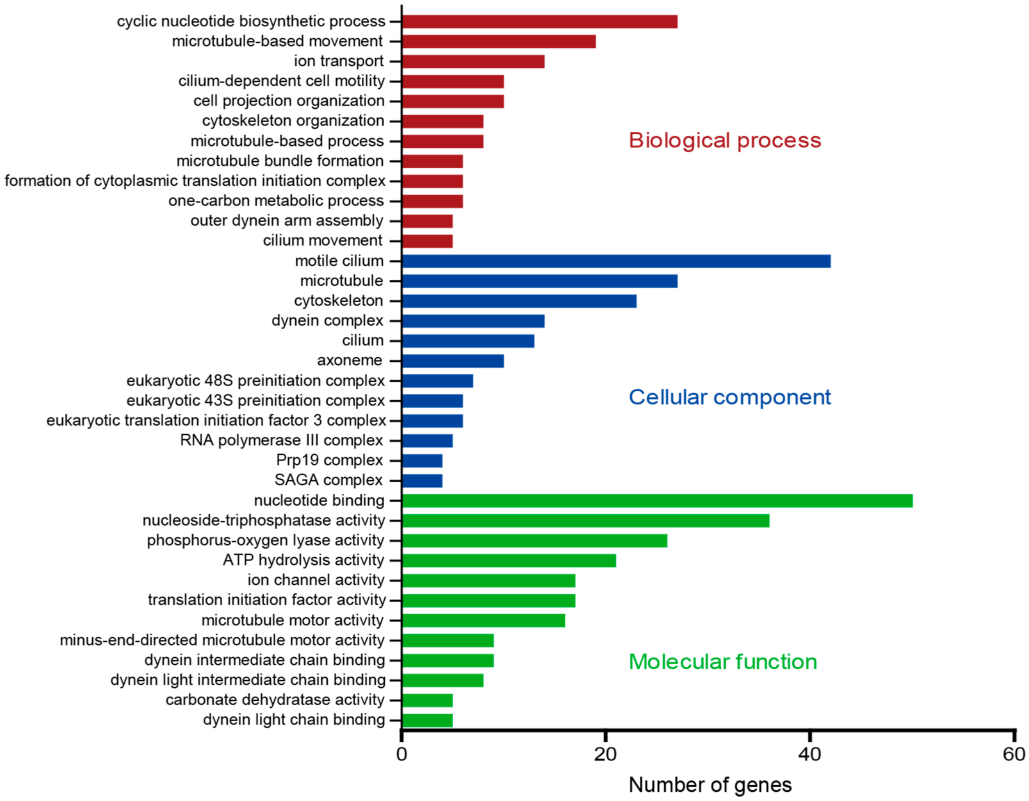

Supplementary Figure S4. GO term enrichment analysis of DEGs in *rus1-1* under cold conditions.

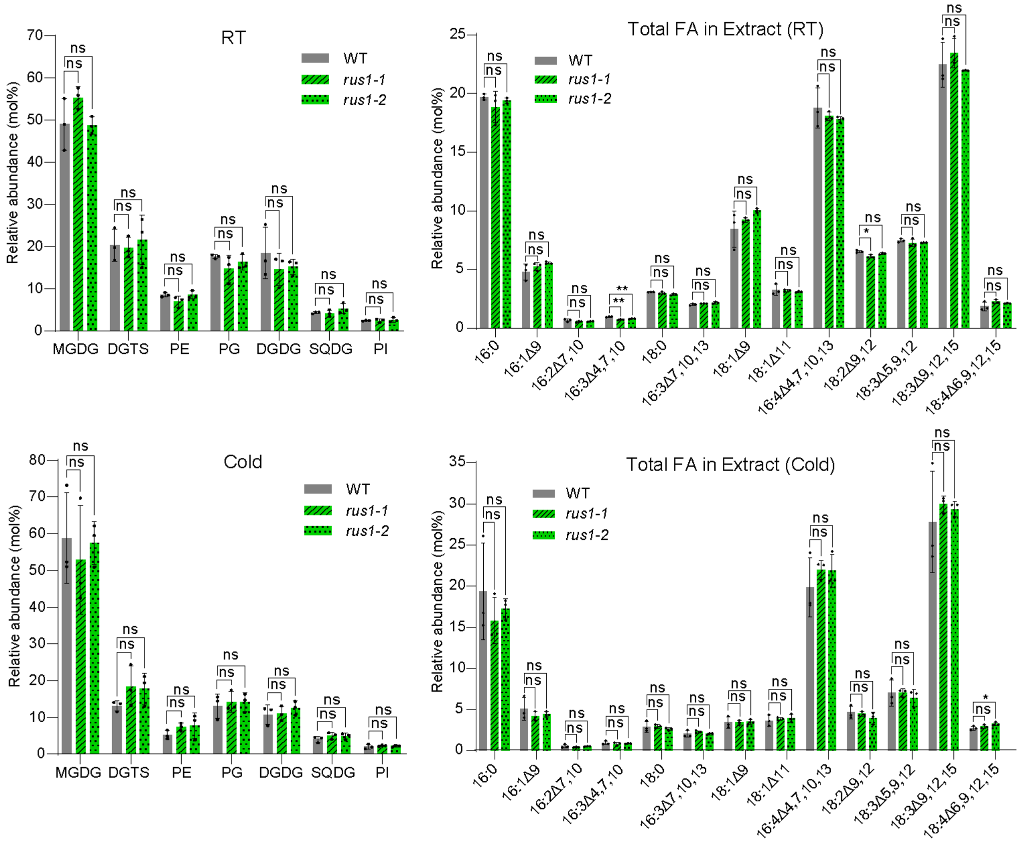

Supplementary Figure S5. Relative abundance of relative fatty acid (FA) composition and major polar lipid classes. For cold treatment, cells were harvested and resuspended in pre-cooled TAP medium at a density of 3×10^6 cells/mL, followed by incubation at 10 °C for 24 h with shaking**.** Asterisks indicate statistically significant differences (*$p < 0.05$, **$p < 0.01$); ns, not significant. Data are presented as the mean ± SEM from three biological replicates.
